# Address by President, A. C. Runyan, D.D.S.

**Published:** 1900-12-15

**Authors:** 


### DENTAL REGISTER.

Vol. LIV. December 15, 1900. No. 12.

### PAPERS READ AT THE UNION MEETING, SEPT. 11 AND 12, 1900, OF THE NORTHERN INDIANA AND THE SOUTHWESTERN MICHIGAN DENTAL ASSOCIATIONS.

### ADDRESS BY PRESIDENT, A. C. RUNYAN, D.D.S.

*Members of the Northern Indiana Dental Society and of the Southwestern Michigan Dental Association:*—It gives me great pleasure as President of the Southwestern Michigan Dental Association, to welcome you all under such favorable auspices; and in behalf of the Southwestern Michigan Association to extend to the Northern Indiana Dental Society, our guests, a most cordial welcome. We trust that the meeting will be as profitable socially and otherwise as we have anticipated, and as we have every reason to expect from the well arranged program and the hearty welcome tendered us by this beautiful city of old St. Joseph by the sea.

Under the circumstances, I think that a brief history of the Southwestern Michigan Dental Association would not be out of place at this time, for it was in this city that the Association came into being.

Briefly its history is this: Through the efforts of Drs.

White and Rockwell, of Benton Harbor, and Drs. Ray and Backus, of St. Joseph, a preliminary meeting was called and a program arranged for the first meeting to be held in the city of St. Joseph, Oct. 8 and 9, 1895. The effort was a success in attendance, quality of its papers and discussions and enthusiasm, and the organization was perfected. As we had fairly good dental laws we desired to have no code of ethics and decided to allow any reputable practitioner, practicing under the laws of the State, to become a member. We have held our meetings semi-annually ever since with increased attendance at each meeting. Feeling so elated over our success and thinking that a little "benevolent assimilation" might be good for all parties, we invited the Northern Indiana Society to meet with us in Benton Harbor, Sept. 14 and 15, 1897. We all recall with pleasure the success of that meeting. The Northern Indiana Society reciprocated with an invitation to meet with them at Elkhart, which meeting was as thoroughly enjoyed. We have had no special place for holding our meetings, but have been fortunate in having so hospitable a membership that each one is always willing to tender the society a cordial invitation to visit his town, and we, as a society, are glad to make St. Joseph twice glad.

Now, the question arises, "What benefits have we received and what good have we accomplished?" By reviewing the past five years we find a few of the benefits that have come to us through the Southwestern Michigan Dental Association. Our State Society meetings were nearly always held in the eastern part of the State, so that, owing to the expense and the time lost from the office, many of us felt that we could not attend. Thus we lost its benefits, and it did not gain our membership. Some of the old State Society members were members of our society, and through them we have been able to get the State meetings nearer the center of the State. At the present time there are few if any members of our society that are not members of the State Society. The last meeting of the State Society, held in Kalamazoo, was one of the largest meetings in its history. Thus we have become acquainted with our fellow-practitioners, and have found them to be as good or better men than ourselves. We have been benefited by their successes, and they have profited by our blunders. Situated as we are so near to our much younger but overgrown sister city, Chicago, we have been benefited at our meetings by listening to papers and witnessing clinics by some of her brightest men in the dental profession. We have been favored by the Michigan State University, being represented by her principal instructors. Who can gainsay that a society or an individual can come in contact with such personalities without being greatly benefited? The proceedings of our meetings have been published in some of the journals, and the hundreds of readers have had the benefits in a smaller way.

While we are congratulating ourselves on what we have done and on the benefits we have received, we should not neglect to bear in mind the fact that there are things that we may and can do for the benefit of the profession.

Our State Society has been a power; in fact, it may be called the father of the dental department of our State University, and we believe our society should be an auxiliary to the State Society in all its undertakings for the upbuilding of the dental profession in our State. And by association with the brethren of Northern Indiana and Illinois we may not only become a power for good in these States, but through the dental journals our influence shall be felt throughout the whole land.

Let us, then, seriously consider our opportunity and make the most of it, that we may develop our own characters and use our talents for the upbuilding of the science and art of our profession.

